# Trajectories of prices in generic drug markets: what can we infer from looking at trajectories rather than average prices?

**DOI:** 10.1186/s13561-022-00384-w

**Published:** 2022-07-11

**Authors:** Antonio J. Trujillo, Jose C. Gutierrez, Emmanuel E. Garcia Morales, Mariana Socal, Jeromie Ballreich, Gerard Anderson

**Affiliations:** 1 Department of International Health, Bloomberg School of Public Health, Johns Hopkins University, Baltimore, USA; 2 Department of Epidemiology, Bloomberg School of Public Health, Johns Hopkins University, Baltimore, USA; 3 Department of Health Policy and Management, Bloomberg School of Public Health, Johns Hopkins University, Baltimore, USA

**Keywords:** Generic drugs, Group based trajectory models, Mergers and acquisitions, Drug, Prices

## Abstract

**Background:**

Well-functioning competitive markets are key to controlling generic drug prices. This is important since over 90% of all drugs sold in the US are generics. Recently, there have been examples of large price increases in the generic market.

**Methods:**

This paper examines price trajectories for generic drugs using a group-based trajectory modelling approach (GBTM). We fit the model using quarterly price information in the IBM MarketScan claims database for the past decade.

**Results:**

We identify three dominant price trajectories for this period: rapid increase trajectories, slow decline and rapid decline. Most generic drugs show a slow or a rapid decline in price trajectories. However, around 17% of all generic drugs show rapid price increase trajectories.

**Conclusions:**

As Congress is exploring an excise tax on drugs whose list price increases faster than the rate of inflation, we discuss what drugs would be most likely to be affected by this law.

## Introduction

The assumption that market forces in the generic drugs industry will discipline firms so that consumers benefit from low prices once a patent expires is increasingly being challenged in the public debate [2, 15]. With low marginal costs of production, and no firms with extraordinary market power due to the homogeneity of products, the conventional wisdom is that generic drug prices should be low and either declining or constant. Yet, data show that for some generics, prices depart from what would be expected in a competitive market [5]. Excessive price increases are considered by policy makers as proof that in some cases, the market fails to provide access to certain drugs at competitive levels [4, 13, 27].

Recent studies have argued for moving beyond headlines of particular drugs to greater understanding of the characteristics of drugs likely to have significant price increases [8, 22]. By looking at price trajectories, and the characteristics of drugs that experience them, we attempt to highlight when market incentives in the generic drugs industry work as expected (i.e. when prices remain constant or decrease) and when current market characteristics fail to discipline prices.

Usually, policy makers focus on static indicators of price changes (i.e., average changes in prices between two specific points in time). We argue that in many instances it could be relevant to explore how the outcome of interest arrives from point A to point B; in other words, the trajectory. This is important in order to understand the market behavior of generic prices. Policy makers aiming to mitigate generic price increases must be equipped to understand how such price increases occur in the current pharmaceutical market. For example, the development of anti-price gouging legislation for generics, such as a 2017 law passed in Maryland, requires that policy makers establish criteria to define what price gouging is. Identifying key trajectories of generic drug pricing behavior may help inform such policies by clarifying the timelines and time frames that can help set policy targets.

Since the enactment of the Hatch-Waxman Act, generic drugs have become increasingly important for consumers’ access to low cost medication [26]. Today, millions of consumers rely on low-price generic drugs for treating a wide range of conditions and in fact nearly 90% of prescriptions dispensed are now generics [7, 16]. The entrance of a generic to compete with a patent protected drug is expected to cause a significant reduction in prices for patients [2, 10]. Furthermore, the level of the price reduction is positively correlated with the entry of additional generic producers in the market [24]. In short, a well-functioning market for generics is designed to maintain access to important drugs at competitive prices [2, 15]. Understanding price dynamics in the generic drugs market is key to evaluating when the market approach is sufficient and when price regulation or some other type of intervention is needed to protect consumers.

We estimate trajectories of generic drug prices over the period 2010 to 2017 using a group-based trajectory modeling approach. We fit the model using quarterly price data for each drug. Our main price outcome is transaction price, which represents the total price the patient and health plan pays to the pharmacy for a prescription. We also estimated the models using monthly price information. As the results were similar, we decided to present the estimates using quarterly price data.

Choosing the appropriate price to compare is important because confidential discounts and rebates may cause prices to vary considerably from published prices such as average wholesale prices (AWP). In addition, out-of-pocket expenditures, coinsurance rates, coupons and patient assistance programs and copayments complicate the interpretations of which final prices are relevant to consider. Lastly, when computing prices, the aggregation of different dosage forms (e.g., tablets, capsules) and strengths (e.g., mg per pill) for the same drug to estimate a single weighted price is another problem that empirical researchers face when analyzing price data of generic drugs.

Fortunately, the IBM MarketScan claims database used in this analysis allows us to minimize these problems. For example, we use a price variable that captures total amount paid per unit of drug during the quarterly period. In section III we provide a detail description of this database and how the data is calculated.

Our methodology allows us to test the existence of three prototypical price trajectories: i) rapid increases in price trajectories, ii) slow increases in prices, and iii) decline in price trajectories. We also explore what drug and market characteristics make a drug more likely to experience a rapid increase in price in comparison to the other trajectories. We then investigate if specific firms are more likely to be associated with generic drugs with rapid price increases.

One primary concern with our approach is the focus on price growth independent of price levels. One may argue that a rational actor would try to get the price right in the initial period and then just grow with inflation. This would still indicate that public health is threatened by these price dynamics even though there may not be any growth to the prices. To partially tackle this issue, we divide all drugs in different groups according to initial price levels. However, we argue that our approach has policy relevance as most public concerns with generic prices are around price dynamics rather than initial price levels.

As most studies of prices, we analyze dynamics of own prices rather than relative prices. One may argue that the price advantage of generic drugs are often viewed in relation to branded drugs. A pattern where a drug grows quickly, but still at a very low level in relation to its brand is different than a drug that is not priced much differently from the brand (still high), but grows more slowly. Policy relevance guide our method as regulators are concern about dynamics of own prices rather than relative prices to a reference brand drug.

Group-based Trajectory model has been applied extensively to study development over time of outcomes in psychology [20], criminology [18], and clinical interventions [1, 9, 11, 21, 29]. Nagin et al. [19] provides a comprehensive review of this method. These types of growth models are used in situations where researchers are interested in studying dynamic paths over time rather than average changes. Section III describes this method in detail.

Our findings suggest that quarterly generic drug price increases observed between 2010 and 2017 can be clustered within three common paths: rapid growth, slow growth and rapid decline in price trajectories. We found that around 17% of generic drugs show a rapid trajectory of price increases. Trajectories of price increases are observed to differ according to level of prices at baseline. High market concentration and low number of competitors are associated with rapid increases in prices. The existence of mergers and high total sale volume of the drugs are not associated with rapid increases in prices. While our findings are consistent with other works that show a general decrease in the price of generic drugs [8, 17, 25], it is true that consistent with other works [5], we were able to identify a group of generic drugs that showed rapid trajectories of price increases.

## Methods

### The economic framework

As mentioned before, one would expect for the prices of generic drugs to act competitively, however, this idea has been challenged recently as the price and market concentration of some generic drugs have increased during the past years [5].

The literature has shown that due to strategic mergers and acquisitions, some pharmaceutical companies had been able to set prices above marginal cost [10, 15]. Similarly, there is evidence suggesting that in markets with few players, a generic firm may collude with other firms to prevent competition [23].

Branded drug companies can also engage in litigation and “pay for delay” strategies which deter or delay a generic firm from entering the market. These actions effectively reduce consumer surplus by preventing competition in the market [2]. In the case of orphan drugs, generic competitors might not enter the market because there is not enough volume. Branded drug companies may restrict supply chain channels, limiting access to the drug by the generic company and thereby avoiding competition [14]. In addition, regulators may prohibit importation from other countries, limiting competition which could help to discipline prices [15]. Lastly, PBMs may distort overall generic drugs prices through the use of confidential price negotiations and the use of spread pricing. In short, numerous factors may distort the competitive functioning of generic drugs market. In all of these cases, prices would show trajectories not consistent with competitive markets. We explore the existence of four theoretical possible developments of prices overtime.

In competitive markets, generic drug companies that increase prices will be driven out of the market by other firms that keep prices closer to marginal costs. We first consider the existence of an upward convexity in the trajectory of prices to determine if drugs with rapid (convex) price increases are more likely to be in highly concentrated markets and in therapeutic areas that have few therapeutic substitutes. In short, “drugs with rapid rising in prices” show a trajectory of rapid price increases which is not consistent with the assumption of competitive markets.

Second, we study the existence of drugs whose prices increase slowly but consistently. The existence of multiple firms should reduce the likelihood of collusive practices [23]. Under this scenario, we test if the market concentration, number of firms and other relevant attributes differ from the first group. The assumption is that drugs “slow rising in prices” have market power but it is less pronounced than the previous group.

We then explore the existence of drugs whose prices decline over time. This path is consistent with the idea that market competition is operating as expected. Generic drug companies are forced to steadily reduce the cost of production and lower prices of generic drugs because of market pressures. Drugs with declining paths are expected to be in markets with large number of providers, several substitutes and low market concentration.

Fourth, we search for the existence of a group of firms that keep prices over time constant. It is expected that marginal cost of production does not change overtime, and price equals constant marginal cost. Firms producing “drugs with stable prices” are under the pressure of competition; but they are able to manage their survival by keeping real prices constant and equal marginal costs rather than reducing them. Adjustments in price setting occurs very rapidly and firms are able to survive by keeping prices constant.

In short, using a Group-Based Trajectory modelling approach, we explore if the quarterly price data for the period 2010–2017 fits the theoretical framework of the existence of these four groups of generic drug price developments: rapid growth, slow growth, decline and steady. It is important to highlight at this point that the GBTM estimates may imply price variations heterogeneity in the drugs markets that it is not consistent with our theoretical framework. Once we evaluate if the dataset fits these patterns, we move to evaluate what market’s characteristics explain paths of rapid increases in prices which are not consistent with the assumption of competitive firms participating in the market for generic drugs. Before outlining the method implemented in this paper, we proceed to explain the price database.

### Dataset

We used the IBM MarketScan Commercial Claims and Encounters database (2010–2017). It includes all claims paid by a selection of around 350 insurers in the USA, representing claims for around 40 million individuals annually. The database contains data on the prices paid by individuals aged 19–64 with employer based health insurance [3].

Most generic drugs are available in different dosage forms (e.g. capsules, tablets, injections) and strengths (e.g. 125 mg, 250 mg, and 500 mg), in addition to being sold by multiple labelers. When we refer to a specific drug, we refer to a specific active ingredient/dosage form/strength combination. For example, Doxycycline, tablet, 500 mg is analyzed as different drug from Doxycycline, tablet, 1000 mg, and these two drugs are analyzed as being in two different markets. Once we clarify how we define our market unit, from this point on we will use the term market and drug interchangeably. Generic drugs are identified using the National Drug Code (NDC) and generic flags included in the main database.

Each drug can be sold by multiple labelers/firms. We consider each one of these labelers as independent players in each market. Although one might be tempted to refer to the labelers as manufacturers, in the pharmaceutical industry, one manufacturer can supply drugs to different labelers. However, our data set allows us to differentiate across labelers, so as it is common in this field, we are going to use number of labelers as number of producers. It is also important to keep in mind that each labeler is a separate company so it is appropriate to treat independently.

Moreover, a single labeler might sell the same product in different package sizes, for example in packages of 50 and 100 pills. As a result, for all the monetary values in the data, we first computed the annual average per-unit price of each drug/ labeler/ package size combination. Then, for each labeler we computed what proportion of its total sales represented each package size. Using this information, we calculated a labeler price index as a sales volume-weighted average of each package size per-unit price.

We included all generic drugs (at the level of dosage form and strength) available in MarketScan for the period 2010–2017. We then restricted our sample to oral solids (i.e., tablets and capsules) according to the criteria layout described above. The final sample comprises 981 generic drugs. We weighted total expenditure data and divided by sales to estimate quarterly weighted prices. We computed average monthly prices and then computed weighted aggregated prices at quarter levels (e.g. Jan-Mar). We ended up with 32 observations per drug.

Once we have the final sample, we classified all generic drugs according to tertiles based on average price across the study period. We dropped the 1% of drugs (9 drugs) with highest observed maximum price which was approximately 15 times the standard deviation in the highest tertile.

Our main price outcome is transaction price, which represents the total price the health plan pays to the pharmacy for a prescription. We use the payment (PAY) variable in MarketScan for the estimation of transaction price. For each drug, the price (PAY) per each strength is a weighted average of all marketed package sizes and their market share. The quarterly price index is a weighted average. All prices are adjusted for inflation using the Medical Care Consumer Price Index.

**Fig. 1:**
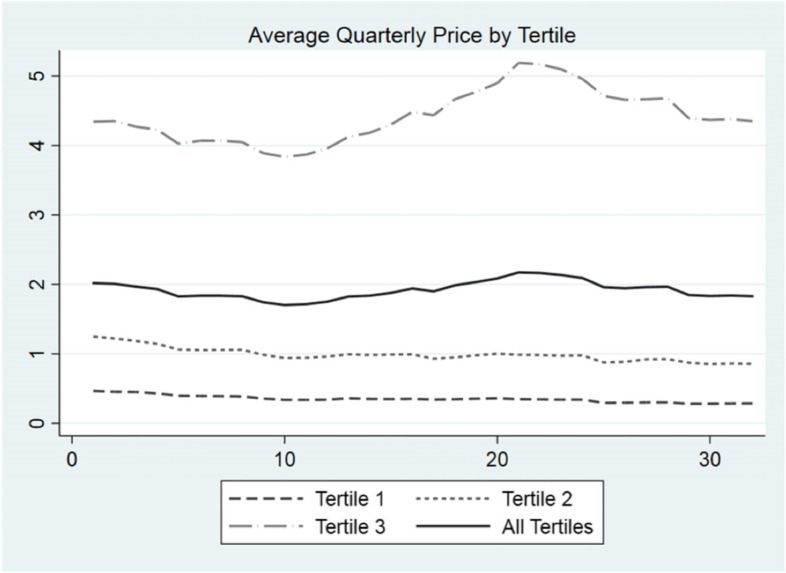
Average Quarterly Prices of Generic Drugs. 2010–2017

Figure 1 displays the evolution of quarterly average price by tertile for the period 2010–2017. Interestingly, the aggregation of each tertile of drugs show stable average real prices with minor upwards fluctuation only in tertile 3. Yet, as we mentioned before, average prices may hide drugs that show rapid price increases or declining price trajectories during the period. Our proposed method aims to unpack the existence of upward price trajectories for certain drugs.

To test robustness of results, we run the analysis using the Average Whole Price (AWP) variable. We understand that AWP is not a good proxy for the prices faced by consumers as rebates and insurers’ payments separate it from the prices faced by consumers. However, it is the price announced by the drug companies and it is often used to determine the cost sharing amount.

### Control variables

Once we identify the relevant paths of price changes, we then investigate the factors that are associated with different rates of growth in price changes. Some control covariates included in the empirical models come from the Red Book database and others from the Marketscan database.

The Red Book database classifies each drug according to their therapeutic group, and if the drug is used to treat acute or chronic conditions. Our therapeutic group variable is a summarized aggregation of the therapeutic group variable in the Red Book database (THERGRP), which originally contained more than 30 categories. Based in part on the CMS categorization of drug classes, we assigned each drug to one of 7 categories: Anti-infective, Cancer, Cardiovascular, Central Nervous System (CNS), Hormones and Synthetic Hormones, Immunosuppressants, and Miscellaneous drugs. The maintenance variable indicates whether a drug is primarily used for short-term treatment of acute conditions, for long-term treatment of chronic conditions, or for both chronic and acute conditions.

The decision to control for differences between acute versus chronic drugs, and controlling for therapeutic groups was driven by previous research that suggests prices and price trends are different across therapeutic classes and acute versus chronic nature of the drugs. One thesis for differences across therapeutic classes and the acute versus chronic nature of drugs is that patients may have different price elasticities based on their condition [6, 25].

We control for baseline prices in 2010 to control for starting point in the path of growth. In order to adjust for the size of the market, we include the total doses prescribed per quarter. We also control for the percentage of the market that it is covered by a brand drug. We consider two drugs to be substitutes if they belong to the same therapeutic class. The existence of a brand drug as a substitute may curtail the capacity of the generic firm to increase prices rapidly. We compute the number of labelers for each generic drug under the assumption that the larger the number of producers, the lower the likelihood that a generic drug shows a path of rapid price increases.

We construct a binary variable that indicates if the generic drug has been impacted by a merger or acquisition (MA). We test if the drugs impacted by MA would be more likely to show a rapid growth in price increases. We compute the Herfindahl-Hirschman Index (HHI) for each drug to assess market concentration. Our original variable is in the scale 0–10,000. Table 1 shows the descriptive statistics for all the variables used in this analysis. Baseline price in the first quarter of 2010 in our sample is around 2 dollars; 10% of all drugs have been impacted by MA, and 25% of all drugs are produced by 10 or more manufacturers.

**Table 1:** Summary Statistics of All Variables

Panel A: *N* = 976	Mean	St. dev.
Baseline Payment (U.S. $)	2.02	3.13
Affected by Merger (%)	0.10	0.30
Average HHI	4839.81	1992.38
Average Doses per quarter (n)	1,345,944.0	3,058,184.0
Average share of branded in market (%)	0.12	0.14
Panel B: *N* = 976	Frequency	%
Number of Labelers (Reference: 0–4) 0–4	318	32.58
5–9	412	42.21
10+	246	25.20
Therapeutic Group (Reference: Miscellaneous) Anti-Infective	113	11.58
Cancer	10	1.02
Cardiovascular	218	22.34
CNS	343	35.14
Hormones	93	9.53
Immunosuppressant	7	0.72
Misc.	192	19.67
Maintenance Group (Reference: Both Chronic and Acute) Both	411	42.11
Prim. Acute	349	35.76
Prim. Chronic	208	21.31
Missing/Other	8	0.82

We included all generic drugs to compute summary statistics. HHI refers to scale 0–10,000

We did not impute values for missing

### The group-based trajectory modelling approach

There are three types of trajectory models [4, 28]: Growth Curve Modelling (GCM), Group Mixture Model (GMM), and Group Based Trajectory Models (GBTM) [19].

All three approaches typically model development over time using different types of polynomials for the variable time (eg. age, quarter, year, etc.), and each method makes different assumptions about the distribution of trajectories in the population of interest, as well as about how to aggregate individual heterogeneity in developments. GCM models the population distribution of individual trajectories and captures mean trends as well as individual departures from the mean trajectory (random effects). GMM is an extension of GCM based on finite mixture models that allows for the presence of multiple GCMs in order to identify the different growth curves of different groups which are considered to be distinct subpopulations with different trajectories. Like GMM, GBTM is also based on finite mixture models, however, the distinction lies in how the subpopulations or trajectories are conceptualized. GMM assumes the existence of specific subpopulations, each with its own GCM, whereas GBTM trajectory groups can be conceptualized as “a statistical device for approximating what is in all likelihood a continuous population distribution of trajectories of unknown shape” [20, 28]. Thus, unlike GCM and GMM, GBTM does not include random effects in the trajectory model.

GBTM models are used when investigators do not have theoretical reasons to hypothesize distinct subgroups with specific trajectories that fit nearly all observations, but rather when seeking to summarize the heterogeneity of trajectories into groups of trajectories that approximate each other. In our case, we hypothesize that prices may follow a few fundamental paths. Therefore, we used the GBTM method to identify the trajectory groups that approximate individual trajectories.

Prior to estimating the trajectory groups, we divided the sample into 3 tertiles based on the average price observed over the study period (2010–2017). Due to the large sample and heterogeneity of trajectories, dividing the 976 into tertiles grouped by price before performing the GBTM analysis allowed the model to converge and produce stable estimates in each tertile.

A key element in the estimation of GBTM is to decide how many groups fit the observed data. In our empirical estimations we use standard statistical tests such as Bayesian information criterion (BIC) to decide the total number of groups to model [12, 19]. However, our theoretical framework suggests the existence of three or four trajectory groups, and this was typically observed in the data as the best-fitting model.

Another challenge in empirical estimations of group-based trajectory modelling is how to handle missing data and outliers [4, 11, 19]. For this analysis, we restricted the sample to 976 generic drugs with observations between quarter 1 of 2010 and quarter 4 of 2017, using the full panel. Fortunately, our price variable does not have missing data problems.

Defining time may be another factor that may influence the estimations of these models [19]. We conducted several robustness analysis. In particular, we conducted robustness tests with price data at yearly and monthly levels. We model the variable time using different types of polynomials, typically including time and time squared, as well as time cubed for some of the trajectory groups. However, it is important to keep in mind that estimating the parameters of these models are computational costly. We tried to approximate the distribution of the data with 2, 3,4, 5 groups. We think three or four groups was the most adequate. We also run several models taking out outliers. Lastly, we computed the groups eliminating 1 year of data at the time to analyze if significant variation in grouping arises as a consequence of eliminating a particular year.

Once we estimate the main price trajectories, we attempt to find predictors of price trajectories. Our selection of predictors was driven by economic theory. Recent empirical work tries to leverage GBTM estimations to have causal interpretation by combining GBTM method with propensity score approach using non-experimental data [18, 21]. In our setting, this option is not possible. Therefore, at this point our predictors of rapid trajectories should be interpreted as important associations which merit further investigation. Finally, to assess what factors may predict a drug having a rising price trajectory, we estimate a logistic regression model using the combined sample for all three tertiles and include the aforementioned covariates in the model.

## Results

### Trajectories of prices in generic drug markets

**Fig. 2:**
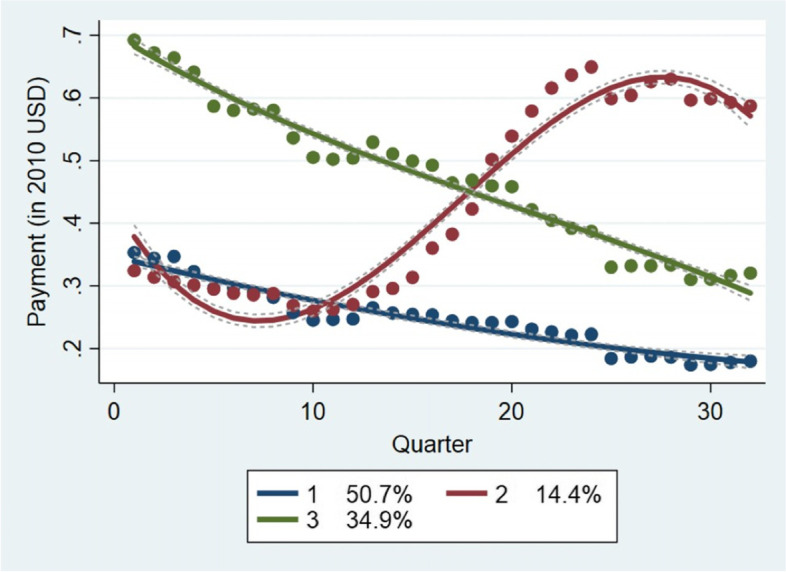
Price Trajectories For Generic Drugs in The First Tertile

Figure 2 displays the trajectories of price changes for generic drugs in the first tertile. The GBTM method indicates that three groups are sufficient to map the topography of price developments among these generic drugs. We estimate that 14.4% of generic drugs in this range of prices show a rapid trajectory of price increases. The rest of the drugs in this price range (85.6%) show declining price trajectories. Yet, around quarter 28, the rapid trajectory in price increases shows a correction downward. The GBTM estimations suggest that 34.9% of the drugs in this price range decline somewhat rapidly while 50.7% decline slowly.

Taken together these results suggests that most low-price generic drugs (at baseline line prices) decline in price over time. For an important proportion of these generic drugs (14.4%), prices increase very rapidly over the period 2010–2017. Competitive forces do not discipline providers of these drugs.

**Fig. 3:**
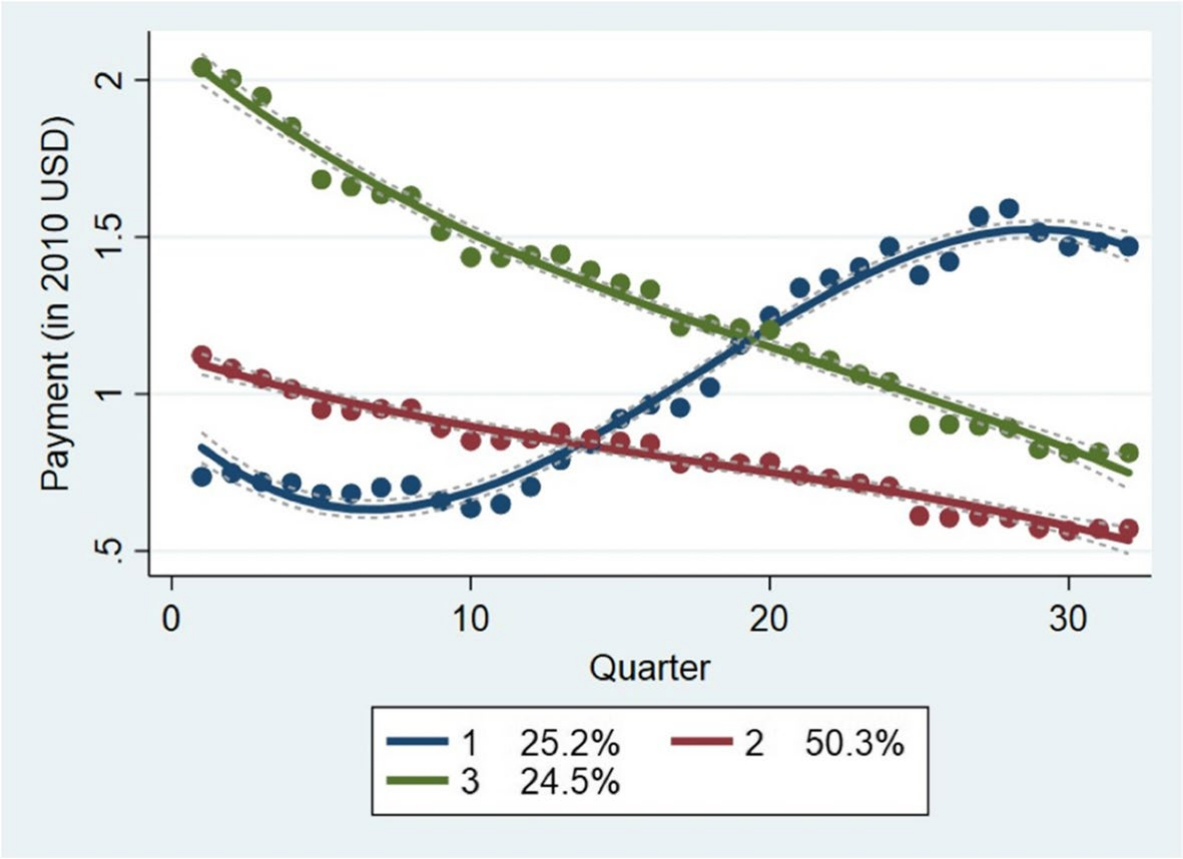
Price Trajectories For Generic Drugs in The Second Tertile

GBTM estimations of the price trajectories for generic drugs in the second tertile indicate that 25.2% of generic drugs in this bracket show rapid increases in price. The remainder of drugs in this tercile show slow decline (Please see Fig. 3).

**Fig. 4:**
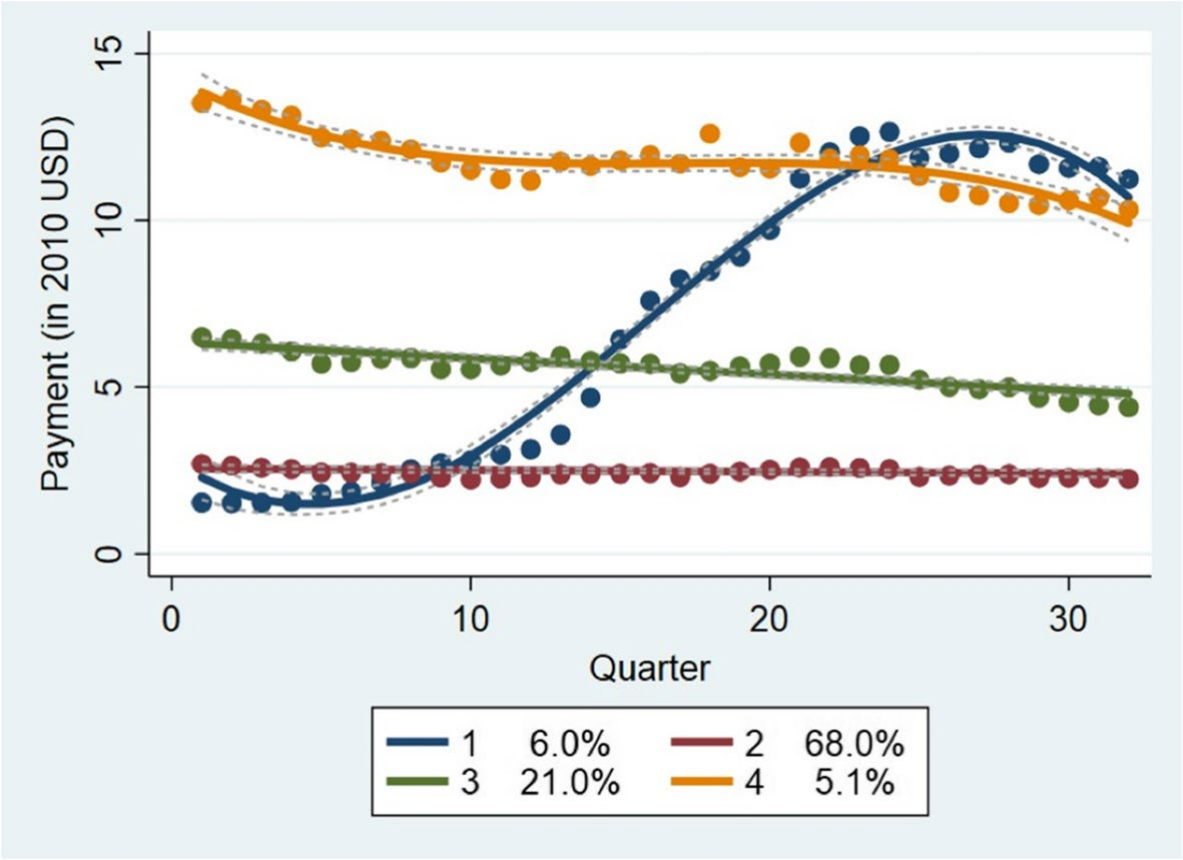
Price Trajectories For Generic Drugs in The Third Tertile

Analyzing high price generic drugs (top tertile), suggests that the topographic of price increases in this price range could be collapsed into four clusters (See Fig. 4). Few drugs show a rapid price increases (6%) while the rest show a steady path in price changes over time. Interestingly, most drugs show constant real prices over time during the period 2010–2017.

In short, for each tertile of generic drugs prices, we find a small cluster of generic drugs that show rapid price increases over the period 2010–2017. These generic drugs are the ones that most likely appear in the headlines and are the poster drugs that suggest to policy makers that market forces are not working [4, 27]. However, our empirical approach shows that most generic drugs can be clustered in slow or rapid decline prices trajectories. These results are consistent with firms experiencing constant marginal costs and prices. Even for the most expensive drugs in these groups, the price trajectories are constant. In short, the GBTM analysis suggests that market competition is working for most of the generic drugs. These results are consistent with the recent findings reported by Frank et al. [8].

### Correlates of a rapid trajectory of price increases

This section studies what predictors are associated with a rapid trajectory of price increases over the period 2010–2017. To investigate this question, we estimated a logit model for the probability of a drug being in the rapid price increase trajectory group. For our estimation we used the covariates mentioned in section III.

We estimated similar probability models for all drugs in each tertile. Table 2 shows the estimated results for the drugs in the first tertile. These results are similar to the findings in the other groups. Results for all tertiles are available in an electronic supplement.

**Table 2:** Logit Estimates of The Associates of a Rapid Trajectory of Price Increases in Generic Drugs. All Drugs

	Coefficient	Std Error
Panel A		
Baseline Payment	−1.414^***^	(0.198)
Affected by Merger	−0.750	(0.506)
Average HHI	0.000256^***^	(0.00001)
Average Doses per quarter	−0.0001	(0.000)
Average share of branded in market	0.925	(0.763)
Panel B		
Number of labelers (Reference: 0–4))		
5–9	−0.206	((0.290)
10+	−1.249^**^	(0.539)
Therapeutic Group (Reference: Miscellaneous)		
Anti-Infective	0.492	(0.483)
Cancer	0.683	(0.922)
Cardiovascular	0.795^**^	(0.373)
CNS	0.467	(0.318)
Hormones	−0.272	(0.444)
Immunosuppressant	2.256	(1.389)
Maintenance Group (Reference: Both Primary and Acute)		
Primarily Acute	0.708^**^	(0.347)
Primarily Chronic	0.474	(0.431)
Constant	−2.036^***^	(0.630)
Log Likelihood	− 307.161	
Pseudo R2	0.255	
Observations	959	

Robust Standard Error in Parentheses, (***) *p* <0.01; (**) *p* <0.05; (*) *p*<0.10

We did not impute values for missing, though there were 9 missing values for the variable Pay

As suggested by our estimates, on average, generic drugs with higher prices at baseline are 19.9% (coefficient − 1.414) less likely to exhibit a rapid increase price trajectory over the period 2010–2017. Also notice that our estimates suggest that drugs produced by a labeler that was involved in a merger, are less likely to be belong to the rapid price increase trajectory group. Contrary to expectations, MAs in generic drugs markets does not seem to increase the market power of firms to set up price increases over time. However, this result is not statistically significant different from zero.

Our estimates show that drugs in highly concentrated markets are more likely to belong to the rapid increase price trajectory. Although highly significant, the magnitude of the effect on the estimated probability is very low. This result is consistent with a situation in which firms in concentrated markets are able to set up rapid price increases for their products as they do not feel the pressure of competitive forces to reduce price increases over time. Similarly, markets with more than 10 providers are 18% (coefficient − 1.249) less likely to show a rapid price increases path (*p* < 0.05). This result suggests that focusing anti-trust regulation on number of players in the market may have an impact on price increases trajectories.

Level of sales as a proxy of relevance of the drug in the market seems not to have an impact on a rapid trajectory of price increases. Markets with higher proportion of brand drugs as substitutes are more likely to show price increases developments; yet, this result is not statistically significant at *p* < 0.10. This might suggest that generic drugs producers who compete with brand drugs are not willing to implement rapid price increases to match brand drug prices.

Another of our hypotheses suggested that generic drugs mainly prescribed for acute care are more likely to be associated with price increases than drugs for preventive diseases because of the differences in demand price elasticities. Accordingly, our estimates suggest that drugs used in acute care are more likely to experience rapid price increases.

### Which drugs are more likely to display a rapid trajectory of price increases?

**Fig. 5:**
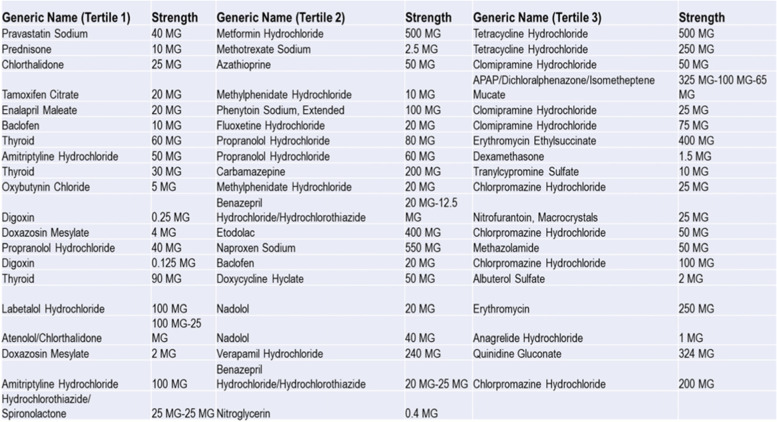
Top 20% Drugs in The Rapid Trajectory of Price Increases by tertile

Figure 5 indicates the names of the best selling drugs (according to baseline sales) in each price tertile that fall into rapid price increase trajectories. By identifying generic drugs with rapid price increases, we validate other research that examined these drugs in isolation and flag these drugs for policy makers who are concerned about public health, access to medications, and the functioning of the generic drug market.

While a variety of drugs are represented in the top 20% of the rapid price trajectory group, some common characteristics exist. Many of these drugs are taken for chronic conditions (e.g. baclofen, thyroid, albuterol). This may support the thesis that companies may set low prices for chronic condition medications to establish themselves in the market. Once established, this forces other manufacturers to drop out from the market so companies can and do pass on price increases reflective of better market power. As previous work indicates that chronic drugs has higher rates of price increases than acute drugs in the branded drug space Schondelmeyer & Purvis [25]; our work suggests this may be true as well in the markets for generic drugs.

The proposed method allows us to identify the list of companies in each tertile that have higher proportion of generic products in the rapid price increases trajectories (results are available upon request from the corresponding author). Companies that are associated with drugs that increase prices consistently may be worthwhile for regulatory examination. The generic market is generally competitive and for the most part prices decline in the long run. While our models do not suggest causation, our results begs the question of why these companies are associated with price increases. It could be the individual drug markets these companies participate in experienced unobserved changes that resulted in price increases or it could be anti-competitive behavior of firms.

## Discussion

Our initial quest to explore price trajectories in generic markets was motivated by policymakers’ concern that market forces are not sufficient to maintain low prices and could affect patient access to these drugs. The group-based trajectory analysis identifies some generic drugs that show a fast pace of price increases over the period 2010–2017. The GBTM estimations indicate that 14.4% of the generics in the low-price range show rapid price increases; while 25.2% of generics in the medium price range and only 6.0% of generics in the high price range show a rapid trajectory of price increases.

The results do not change when we run the models with monthly or yearly prices. Furthermore, when we use the variable AWP the price trajectories estimations for each tertile do not change. In short, we feel confident that our estimates are robust to a battery of relevant model and data specifications. Taken together our estimations suggest that the application of the GBTM models in this setting seem to fit the observed heterogeneity in price changes well.

It appears that market forces are constraining price increase trajectories in most generic drugs. Our results are in line with findings reported by [8]; yet we focus on price trajectories rather than changes in average annual prices. We argue that this method provides a more comprehensive picture of the markets’ performance.

Our results indicate that a larger number of players in the market (+ 10) are linked to a decline in the probability of having upward trajectories in prices. Market competition seems to discipline prices when there is robust competition. As expected, high market concentration plays a role in putting a product in a rapid trajectory of price increases. Drugs that are already expensive are less likely to exhibit upward price trajectories. Acute treatments are more likely to be on the upward path. One possible explanation is that acute treatments rely on new users, who may not know about the previous lower prices.

## Conclusions

The paper has interesting and important ambitions. First, it describes price trajectories of generics, and then, identifies the characteristics of generic drugs that experience the biggest price increases. Describing the trajectories has been done in other papers using average prices; yet, we propose an alternative approach such as Group Based Trajectory Model which provides a comprehensive mapping of price changes. We fit the model using quarterly price information in the IBM MarketScan claims database for the period 2010–2017. We identify three dominant price trajectories for this period: rapid increase trajectories, slow decline and rapid decline. Most generic drugs show a slow or a rapid decline in price trajectories. Around 17 % of all generic drugs show rapid price increase trajectories. The identification of characteristics of generic drugs with the biggest price increases is an important contribution with relevant policy implications. For instance, drug pricing reforms that plan to penalize pharmaceutical companies for unsupported price increases may based financial penalties on trajectories of prices over time rather than on a ceiling related to inflation rate for the average price increases during a specific period.

Importantly, the approach that we propose identifies the drugs that follow a path of rapid price increases. This information would be valuable for state and federal regulators to moitor price increases, and for an anti-trust agency to monitor anti-competitive behavior of firms in these markets. The method could be used to monitor those companies who are in a trajectory of rapid price increases, or to monitor those drugs that went off the trajectory of constant prices towards a path of more rapid increases.

In addition, this method permits the identification of factors that push drugs into rapid price increase trajectories. This information may be used preemptively by anti-trust agency to monitor competition and protect consumers’ welfare. Future research should study if product-based regulation according to price trajectories may enhance consumers’ welfare more than attaching price increases for all generic drugs to annual inflation rate. This is an important factor to weigh in the policy debate because an important proportion of generic drugs exhibit declining price trajectories over time. Future research should explore how often the market corrects for over-pricing behavior, and how long it takes to do so. Lastly, future researchers should incorporate in a structural model how dynamic changes in some of the control variables are linked to changes in drug prices.

## Data Availability

This paper only uses public available data sets. The full analysis can be replicated having access to the IBM MarketScan claims for the period of the study.

## References

[1] Allen NB, Siddique J, Wilkins JT, Shay C, Lewis CE, Goff DC, Jacobs DR, Liu K, Lloyd-Jones D (2014). Blood pressure trajectories in early adulthood and subclinical atherosclerosis in middle age. Jama.

[2] Berndt ER, Conti RM, Murphy SJ (2017). The landscape of us generic prescription drug markets, 2004–2016.

[3] Blewett LA, Call KT, Turner J, Hest R (2018). Data resources for conducting health services and policy research. Annu Rev Public Health.

[4] Cole AL, Sanoff HK, Dusetzina SB (2017). Possible insufficiency of generic price competition to contain prices for orally administered anticancer therapies. JAMA Intern Med.

[5] Dave CV, Kesselheim AS, Fox ER, Qiu P, Hartzema A (2017). High generic drug prices and market competition: a retrospective cohort study. Ann Intern Med.

[6] DiMasi JA (2000). Price trends for prescription pharmaceuticals: 1995–1999. Department of Health and Human Services Conference on pharmaceutical pricing practices, utilization, and costs.

[7] Federman AD, Halm EA, Zhu C, Hochman T, Siu AL (2006). Association of income and prescription drug coverage with generic medication use among older adults with hypertension. Am J Manag Care.

[8] Frank RG, Hicks A, Berndt ER (2019). The price to consumers of generic pharmaceuticals: beyond the headlines.

[9] Franklin JM, Shrank WH, Pakes J, Sanfélix-Gimeno G, Matlin OS, Brennan TA, Choudhry NK (2013). Group-based trajectory models: a new approach to classifying and predicting long-term medication adherence. Med Care.

[10] Hernandez I, Good CB, Cutler DM, Gellad WF, Parekh N, Shrank WH (2019). The contribution of new product entry versus existing product inflation in the rising costs of drugs. Health Aff.

[11] Hu X, Gu S, Sun X, Gu Y, Zhen X, Li Y, Huang M, Wei J, Dong H (2019). Cognitive ageing trajectories and mortality of chinese oldest-old. Arch Gerontol Geriatr.

[12] Jones BL, Nagin DS (2013). A note on a stata plugin for estimating group-based trajectory models. Sociol Methods Res.

[13] Joyce G, Henkhaus LE, Gascue L, Zissimopoulos J (2018). Generic drug price hikes and out-of-pocket spending for medicare beneficiaries. Health Aff.

[14] Karas L, Shermock KM, Proctor C, Socal M, Anderson GF (2018). Limited distribution networks stifle competition in the generic and biosimilar drug industries. Am J Manag Care.

[15] Lakdawalla DN (2018). Economics of the pharmaceutical industry. J Econ Lit.

[16] Mahoney JJ (2005). Reducing patient drug acquisition costs can lower diabetes health claims. Am J Manag Care.

[17] Mandel M (2019). The prescription escalator: the real reason why americans pay more for drugs each year, why they are so upset and what can be done about it.

[18] Nagin DS (2016). Group-based trajectory modeling and criminal career research. J Res Crime Delinquency.

[19] Nagin DS, Nagin D, et al. Group-based modeling of development. Harvard University Press; 2005.

[20] Nagin DS, Odgers CL (2010). Group-based trajectory modeling in clinical research. Annu Rev Clin Psychol.

[21] Nagin DS, Tremblay RE (2005). What has been learned from group-based trajectory modeling? Examples from physical aggression and other problem behaviors. Ann Am Acad Pol Soc Sci.

[22] Part D (2016). Generic drug prices declined overall, but some had extraordinary price increases.

[23] Porter RH (2005). Detecting collusion. Rev Industrial Organization.

[24] Reiffen D, Ward MR (2005). Generic drug industry dynamics. Rev Econ Stat.

[25] Schondelmeyer SW, Purvis L (2016). Trends in retail prices of brand name prescription drugs widely used by older americans, 2006 to 2015. Rx Price watch report.

[26] Selden TM, Abdus S, Miller GE. Decomposing changes in the growth of us prescription drug use and expenditures, 1999-2016. Health Serv Res. 2019;54(4):752-63. 10.1111/1475-6773.13164. Epub 2019 May 9. 10.1111/1475-6773.13164 PMC6606541 31070264

[27] Shakil S, Redberg RF (2017). New (very high) prices on old drugs. JAMA Intern Med.

[28] Warren JR, Luo L, Halpern-Manners A, Raymo JM, Palloni A (2015). Do different methods for modeling age-graded trajectories yield consistent and valid results?. Am J Sociol.

[29] Zimmer Z, Martin LG, Nagin DS, Jones BL (2012). Modeling disability trajectories and mortality of the oldest-old in China. Demography.

